# Supplementary material for: Effects of Tetranychus urticae infection on phyllosphere microbial community assembly of Vigna unguiculata
Source: PeerJ. 2025 Dec 1;13:e20389. doi: 10.7717/peerj.20389 (PMC12677042; doi:10.7717/peerj.20389)
Supplement: Supplemental Information 1 — a: The number of connections obtained by SparCC correlations. b: Microbial taxon (at ASV level) with at least one significant (P < 0.05) and strong (SparCC > 0.7 or < −0.7) correlation c: SparCC positive correlation (> 0.7 with P < 0.05) d: SparCC negative correlation (< −0.7 with P < 0.05) e: The node connectivity, that is, the average number of connections per node in the network. HE, UHE represent e ndophytes in the uninfected and infected leaves of V. unguiculata, respectively; HA, UHA represent the epiphyte in the uninfected and infected leaves of V. unguiculata, respectively. [file peerj-13-20389-s001.docx]

Table S1 Topological properties of microbial networks

|  | Bacteria | | Fungi | |
| --- | --- | --- | --- | --- |
|  | UHE and UHA | HE and HA | UHE and UHA | HE and HA |
| Number of edges^a^ | 358 | 817 | 201 | 309 |
| Number of nodes^b^ | 95 | 98 | 60 | 64 |
| Positive edges^c^ | 323 | 741 | 174 | 261 |
| Negative edges^d^ | 35 | 76 | 27 | 48 |
| Average degree^e^ | 7.537 | 16.673 | 6.700 | 9.656 |
