# Supplementary material for: Effects of Tetranychus urticae infection on phyllosphere microbial community assembly of Vigna unguiculata
Source: PeerJ. 2025 Dec 1;13:e20389. doi: 10.7717/peerj.20389 (PMC12677042; doi:10.7717/peerj.20389)
Supplement: Supplemental Information 2 — The sum of the proportions of Deterministic (%) and Stochastic (%) is 100%. Whichever process exceeds 50% for a sample is the dominant process for that sample. HE, UHE represent endophytes in the uninfected and infected leaves of V. unguiculata, respectively; HA, UHA represent the epiphyte in the uninfected and infected leaves of V. unguiculata, respectively. [file peerj-13-20389-s002.docx]

Table S2 Relative contribution of components in the assembly process of bacterial and fungal communities

|  | Sample | Deterministic (%) | Stochastic (%) |
| --- | --- | --- | --- |
| bacteria | HE | 8.7 | 91.3 |
|  | UHE | 51.5 | 48.5 |
|  | HA | 38.7 | 61.3 |
|  | UHA | 76.4 | 23.6 |
| fungi | HE | 78.2 | 21.8 |
|  | UHE | 49.7 | 50.3 |
|  | HA | 77.9 | 22.1 |
|  | UHA | 25.3 | 74.7 |
