# Supplementary figures and images for: Effects of Tetranychus urticae infection on phyllosphere microbial community assembly of Vigna unguiculata
Source: PeerJ. 2025 Dec 1;13:e20389. doi: 10.7717/peerj.20389 (PMC12677042; doi:10.7717/peerj.20389)

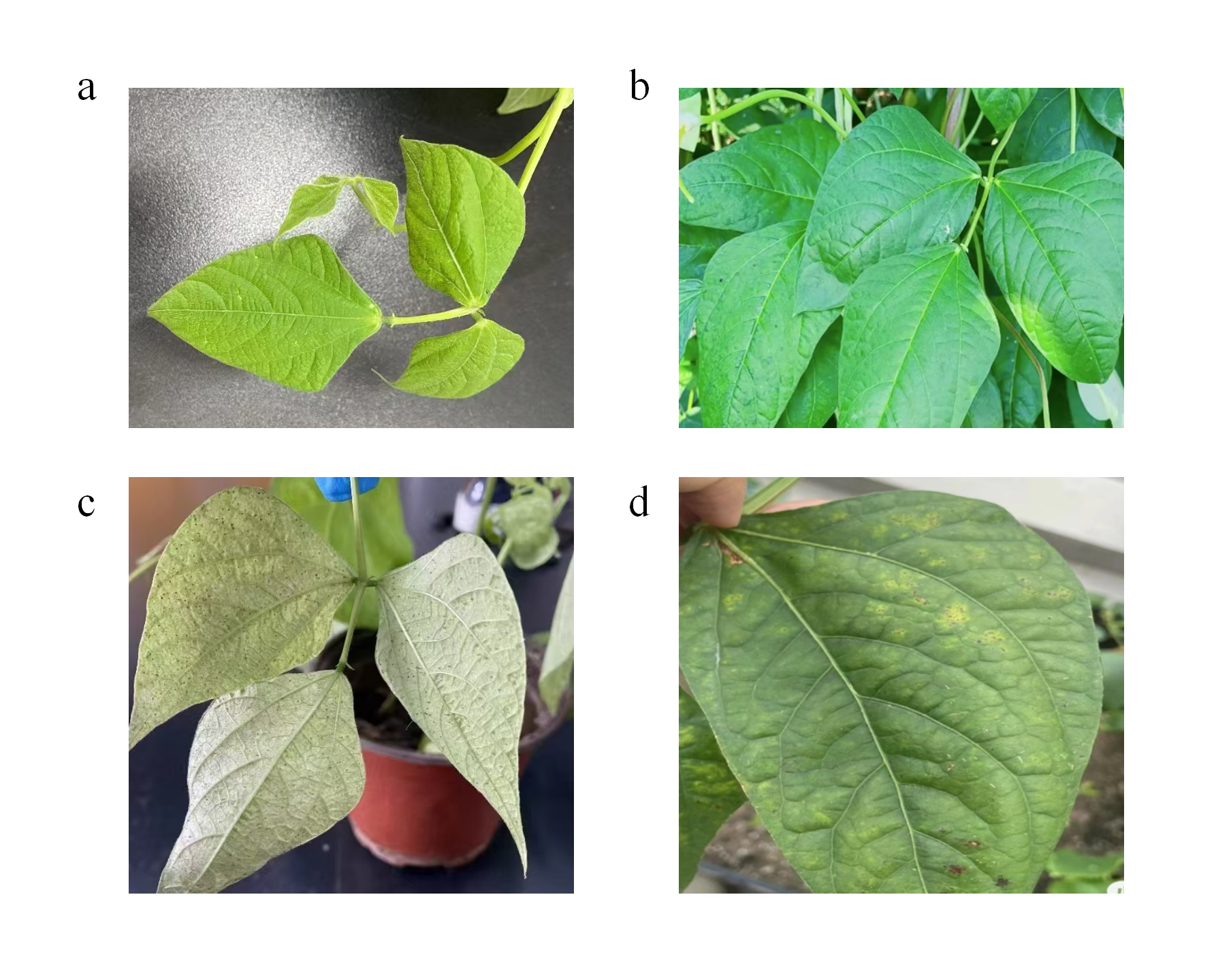

Supplement: Supplemental Information 3 — Note: (a) and (b) uninfected phyllosphere of Vigna unguiculata, (c) phyllosphere of Vigna unguiculata infected by Tetranychus urticae, (d) leaf morphology of Vigna unguiculata after infection. [file peerj-13-20389-s003.png]

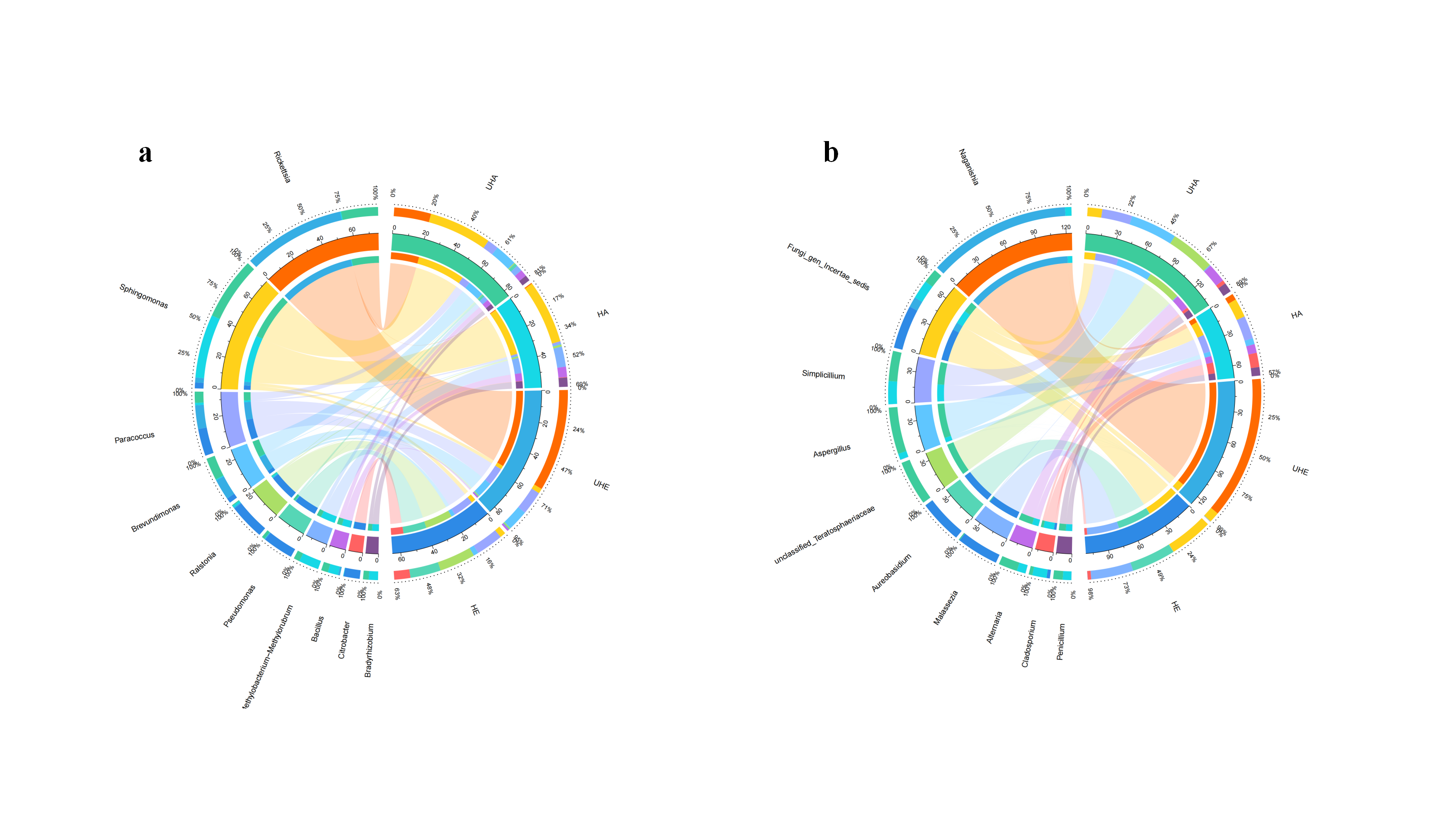

Supplement: Supplemental Information 4 — (a) the dominant phyllosphere bacterial genera, (b) the dominant phyllosphere fungal genera. The left half circle represents the dominant genera, and proportions of each genus in different samples. The right half circle represents the different samples. HE, UHE represent endophytes in the uninfected and infected leaves of V. unguiculata, respectively; HA, UHA represent the epiphyte in the uninfected and infected leaves of V. unguiculata, respectively. [file peerj-13-20389-s004.png]
